# Strangulated Hernia

**Published:** 1894-07

**Authors:** 


STRANGULATED HERNIA.—Gay (*Boston Medical and Surgical Journal*, December 28, 1893) gives some cases which he hopes may direct attention to the importance of giving these cases *early* relief. The vitality of the patient is often much more exhausted than is indicated to the ordinary observer by the general symptoms and appearance. The first patient, a man, aged thirty-seven years, had a "compound" hernia. He walked into the accident room, let down his trousers, and showed a portion of the intestines protuding through the skin. The accident had occurred while lifting, twenty-four hours previously, and the protruding intestine, four inches in length, was covered with a thick layer of dirty grayish lymph. There was no sign of strangulation. The case was operated on and the intestines replaced. Suppuration occurred, and convalescence, while slow, was satisfactory. The second case, a woman, aged fifty-two years, had a strangulated hernia of a week's standing. There was vomiting, constipation, and pain. The vomiting was stercoraceous. Three and a half inches of the intestines were found to be purple, thickened, and not glistening. It was excised and end to end coaptation done with two rows of Lembert's sutures. Everything went well till the ninth day, when peritonitis set in and she died. No autopsy. Another case was that of a man, aged fifty-six years, operated on in collapse. A very little ether was given, and the bowel found to be covered with thick lymph, threatening sloughing. The constriction was divided, the bowels placed just inside the wound, and the latter packed with baked gauze. The wound was closed with suture in five days, and healed in five weeks. In three cases of gangrenous hernia, operated on while in collapse, the bowel was deliberately opened with a stroke of the knife, and the constriction divided by carrying the herniotomy into the lumen of the intestine. Two of these lived a fortnight, and the third recovered. Four additional cases are detailed in which the constriction was above the internal ring. If suspected rupture is accompanied by vomiting, operation is, as a rule, demanded. Save time, avoid shock, preserve the animal heat, and keep the body dry.—*University Medical Magazine.*

